# Mediating Role of Optimism Bias and Risk Perception Between Emotional Intelligence and Decision-Making: A Serial Mediation Model

**DOI:** 10.3389/fpsyg.2022.914649

**Published:** 2022-06-03

**Authors:** Chaoran Chen, Muhammad Ishfaq, Farzana Ashraf, Ayesha Sarfaraz, Kan Wang

**Affiliations:** ^1^School of Economics, Liaoning University, Shenyang, China; ^2^Department of Management Sciences, Riphah International University, Faisalabad, Pakistan; ^3^Department of Humanities, COMSATS University Islamabad, Lahore, Pakistan; ^4^Department of Human Development, University of Home Economics Lahore, Lahore, Pakistan; ^5^School of Software, Henan University, Kaifeng, China

**Keywords:** emotional intelligence, optimism bias, risk perception, investment decisions, commodity market

## Abstract

The commodity market plays a vital role in boosting the economy. Investors make decisions based on market knowledge and ignore cognitive biases. These cognitive biases or judgment errors have a significant effect on investment decisions. Therefore, this study aimed to investigate the effect of emotional intelligence on decision-making. In addition, optimism bias and risk perception are the intervening variables between emotional intelligence and decision-making. So, this study contributes to the body of knowledge by examining the mediating role of optimism bias and risk perception. The data were collected from the respondents of the commodity market and the 370 questionnaires were distributed among the investors, of which 337 respondents gave their feedback. The convenience base sampling technique is used due to the easy access of respondents, time factor, and cost factor. Data entered into the SPSS Statistics Version 26 and PROCESS macro model 6 were used for serial mediation. AMOS was used for the validity and model-fit analysis. The results of this study aligned with the literature that there is a significant effect of emotional intelligence on decision-making. It also observed that optimism bias has a positive effect on decision-making. The finding of this study will be helpful for the brokers, the government, and especially the investors. This study also proposed that future studies on the stock exchange and real estate market comparative analysis can be conducted.

## Introduction

Over time, the paradigm of behavioral finance has moved the attention away from traditional finance. According to the classical financial paradigm, researchers explain investors’ decisions by focusing on behavioral finance reactions (Sartori et al., 2017). Behavioral finance focuses on judgment errors that deal with the irrational behavior of investors. Investors have other mental biases that can severely prevent them from growing their wealth (Shefrin and Statman, 2000). In traditional finance, decision-makers estimate all possible results that are presented as the foundation for creating the conventional prospect in finance. Finance speculations assume that these rational individuals are unwilling to take the risk (Jackson and Jabbie, 2019). Traditional decision theory believes that individuals are the logical decision-makers who are self-seekers in the existence of restraints.

Behavioral finance focuses on how the investor’s psychology influences the decisions that may be positively or negatively (Zhuo et al., 2021). In behavioral finance, investors make decisions based on emotions and cognitive biases. The study of psychology that influences investor’s decisions and the marketplace is known as behavioral finance (Shefrin and Statman, 2000). According to the behavioral finance models, rather than just market data, individual personality, thinking, emotions, and judgment errors are all affected by investment decisions. Investors’ actions are not necessarily rational; they can be motivated by psychological or attitudinal factors.

Salovey and Mayer (1990) proposed the “Emotional Intelligence” concept. Emotional intelligence is referred to as the use of information to influence one’s thinking and behavior and the ability to be aware of one’s feelings (Salovey and Mayer, 1990). A five-dimensional emotional intelligence model is proposed by Goleman (1998): social skills, empathy, motivation, self-regulation, and self-awareness, all of which are necessary qualities (Goleman, 1998). The researchers looked into how incidental emotions differ from scenario to circumstance and how they influence decisions about the normative approach, which is artificial to that mood or feeling (Campos and Keltner, 2014). The trip of an ordered reaction that crosses the subsystems of psychology, motivational, experimental, and cognition, is referred to as emotion.

Investors’ judgments are influenced by cognitive bias, which is a systematic inaccuracy in thinking. Some of these biases are memory related; how you recall an event might be skewed for various reasons, resulting in judgment errors and decision-making errors. On the other hand, cognitive biases are linked to the roles performed by different types of experience and knowledge (Bodnaruk and Simonov, 2015). Psychological feelings have been defined as a component of perception in general. As a result, they are inescapable and can be found in many settings and assignments (Murata et al., 2015). According to the researchers, the rationality of investors is severely limited. Simon (1955) coined the term “bounded rationality” to describe his skepticism of anticipated utility theory’s assumption that decision-makers are fully rational (Von Neumann and Morgenstern, 1947).

Investors who think positively always act optimistically and become inspired to invest (Anderson and Galinsky, 2006). On the other hand, the less motivated investor always makes decisions based on market trends, and if the investor believes the market is performing well, they will invest. When a speculator constructs his venture/capital formation, he is idealistic in light of reality and believes that future outcomes will occur without fail (Galanti and Gaël, 2017). When investors are optimistic, they put their money into portfolios to maximize their profits (Moamen et al., 2016).

Risk perception is a critical element in decision-making (Singh and Bhowal, 2010). Risk is a complex and important factor (Cristofaro, 2018). Values are preferred by speculators with a high level of budgetary proficiency, while bank shops are preferred by financial specialists with a low level of money-related knowledge. Furthermore, as mentioned in the literature, men have a higher level of advanced financial education than women. As per the chance of profit or loss, risk perceptions are considered. The severity of individuals and risk features are measured by risk perception. Risk perception is a decision-making process that is based on an individual’s lifetime frame of reference, among other things (Robinson and Marino, 2015).

Commodity exchanges are located in many nations around the world, and they serve as a marketplace for sellers and buyers to trade commodities. Mercantile exchange is a frontier market that deals with four key assets: metals, agriculture, energy, and financial futures (Nguyen and Rozsa, 2019). Dutta et al. (2017) investigated the existence of uncertainty in the financial market and how this uncertainty affects the commodity market (Limongi and Ravazzolo, 2019). Inoue and Hamori (2014) also examined the efficiency of the market with respect to commodities’ future market and how the future market affect the prices of shares.

According to the prospect theory (Tversky and Kahneman, 1992), rather than a perceived risk of loss, investors prefer to make decisions based on the perceived possibility for gain when an outcome is uncertain (Baker et al., 2019). Investors behave rationally and use all the available information. Drawing mainly from the prospect theory, this study fills the gap by studying the effect of emotional intelligence on the decision-making. Moreover, considering the lack of research on the impact of cognitive bias on risk perception, this study contributes to the body of knowledge by investigating the effect of optimism bias on risk perception and decision-making. Risk perception refers to a subjective judgment that deals with individuals’ perception of the severity of a risk. Ishfaq et al. (2020) also examine the mediating effect of the risk perception between cognitive biases and investment decisions. Simon et al. (2000) also investigated that risk perception mediates the relationship between cognitive biases and the decision to start a venture. Keeping in view the recent studies, the objective of this study is to investigate the effect of emotional intelligence on decision-making with the mediating effect of optimism bias. So, the research question is also aligned with the objective of this study: “How emotional intelligence affects decision-making *via* the mediating effect of optimism bias and risk perception.”

## Literature Review

### Theoretical Background

#### Prospect Theory

Expected utility theory states that investors are included in decision-making, compared with their expected utility value, and distinguishes between risk and uncertain consequences. Tversky and Kahneman (1989) proposed an alternative model (prospect theory) because the expected utility theory model is an expressive decision-making model under risk. An alternative model can clarify the risk in multiple outcomes. The prospect theory focuses on the potential outcome before attaining a result. For explaining the investors’ decision, literature supported that investors’ judgment errors, emotions, and personalities reflect the decision outcome. As the prospect theory is based on the uncertainty factor (risk) and the judgment errors (preferred to gain profit even it is nominal), so based on this theory, hypotheses are generated by focusing on the emotional intelligence, optimism bias (judgment error), and risk perception. Investors usually make decisions based on possible losses/gains, and when the same selections are available in various forms, their preferences are unpredictable. According to a prospect theory by Tversky and Kahneman (1989), losses are associated with profits due to investors’ common and irrational propensity. In a speculation market, loss is nominal, and investors act in moving toward profits more than losses.

### Hypotheses Development

#### Emotional Intelligence and Decision-Making

In the way how the individuals think, carry on, and make investment decisions, it has been stated that emotions are unpredictably bound up in financial markets. The capacity to know about one’s own feelings and others’ feelings is referred to as emotional intelligence (Brettschneider et al., 2021). The effect of emotions on the decision-making process is supported by many significant investigations (Mitchell et al., 2019). Emotional intelligence can comprehend the components, how they fluctuate, and therefore see about feelings; it is also used to handle the issue and recognize one’s own and others’ emotions. In the decision-making process, emotional intelligence is a significant determinant.

**H1:** Emotional intelligence has a significant effect on investment decisions.

#### Risk Perception and Decision-Making

Risk perception is an influencing variable and works as an intervening variable in the literature. Moreover, Ishfaq et al. (2020) also examined the mediating role of risk perception between cognitive biases and investment decisions. Simon et al. (2000) also reported risk perception as an intervening variable in the capital and venture formation business. They also mentioned that other influence factors like cognitive biases should be examined and their effect must be checked on the decision-making. Nguyen and Rozsa (2019) examined that risk factors (perception and tolerance) significantly impact investment intentions. Cognitive biases or judgment errors significantly affect investment decisions (Ishfaq et al., 2020). In addition, a partial effect is examined between the behavioral biases and investment intentions through the mediating effect of risk perception. Risk perception shows a generative mechanism through which cognitive biases influence decision-making (Pandey and Jessica, 2019).

**H2:** Risk perception has a significant effect on decision-making.

#### Optimism Bias and Decision-Making

Most of the time, individuals invest in stock markets to earn, but most people do not know that the choice to invest in those stocks is affected by traditional and behavioral finance. There is the presumption that the investors are much more rational in conventional finance. They assemble or get all the data they require, and their choices are based on that information (Trevelyan, 2008). In this manner, traditional finance essentially states that investors do not make financial choices based on their personal feelings and emotions. On the other hand, behavioral finance clarifies that individuals are optimistic and unreasonable and their feelings play part while making investment choices (Rasheed et al., 2018). Several efforts were made to understand which factor primarily influenced the individual’s decision-making process. Decision-making is the most complicated task for investors when investing in financial markets.

**H3:** Optimism bias has a significant effect on decision-making.

#### Emotional Intelligence and Optimism Bias

Hopeful individuals could not care about the complex situation as they can undoubtedly face problems. When people are approached to anticipate constructive and pessimistic occasions, they are strikingly biased and remark in a specific heading. Whether circumstances are sure or negative, bias can develop contingent (Siegrist, 2021). To control a person’s emotional life and achievement, the idea of emotional intelligence is considered. Emotional intelligence is characterized as sorting out life to advance it, creating sympathy with others, and a person getting together his sentiments.

Under five fundamental themes, Goleman (1998) recorded intelligence. Emotional intelligence and social aptitude are critically connected. People can adjust with their present circumstances, defeat more issues very effectively, and have an emotional state of intelligence. In this manner, people feel less focused when they are facing distressing circumstances while having abnormal emotional intelligence (George and Mallery, 1999).

**H4:** Emotional intelligence has a significant effect on optimism bias.

#### Emotional Intelligence and Risk Perception

Ingram et al. (2019) stated that emotional intelligence (EI) has two distinct methodologies: character quality and various emotional preparation abilities. Social intelligence is defined as understanding the gender (men and women) to act wisely in their relations. Salovey and Mayer (1990) first defined emotional intelligence as “the ability to monitor one’s own and others’ feelings and emotions, discriminate among them, and use this information to guide one’s thinking and action.” From their original definition, Salovey and Mayer (1990) investigated that emotional intelligence consists of self-emotional appraisal, other emotional appraisals, and regulation and use of emotions.

**H5:** Emotional intelligence has a significant effect on risk perception.

#### Optimism Bias and Risk Perception

Financial experts at institutions have a good sense of whether the economy is in a good place. For example, if stock exchanges are dependable and institutional financial specialists’ customers gain, they may be less likely to lose their positions, even if their exhibition lags market returns. Institutional financial investors may have a reason to be optimistic about future economic conditions. One of the features of constrained rationality is information selection bias, which leads to optimism (Simon et al., 2000). People tend to be optimistic about the future, underestimating the likelihood of bad events while overestimating the probability of positive events. When a person feels that they are less likely than others to experience a negative occurrence, optimism bias occurs (Suchanek, 2021). Optimism bias is commonly quantified using risk determinants (Neal et al., 2022). Individuals are asked to evaluate their chances of experiencing a negative event vs. another person’s options of sharing the same unfavorable occurrence (Liu et al., 2018).

**H6:** Optimism bias has a significant effect on risk perception.

**H7:** Optimism bias and risk perception mediate the relationship between emotional intelligence and investment decision.

**FIGURE 1:**
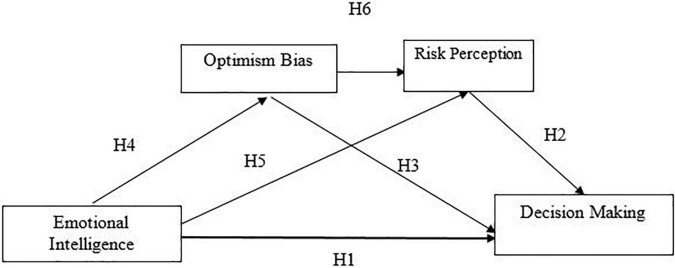
Conceptual framework.

Political conditions, market information, and rumors impact the investor’s decision and the volume of shares traded differently. So, the anomalies and biases may also affect the investment decisions. Optimism bias and risk perception related to the investment align with the prospect theory and significantly affect investment decisions. This conceptual framework (Figure 1) depicts that emotional intelligence affects decision-making *via* the mediating role of optimism bias and risk perception.

## Method and Analysis

### Target Population and Sample Size

Ordinary people make irrational decisions based on judgment errors. The commodity market is the most influential and significantly affects investment decisions. The target market of this study is the individual investors of the commodity market. The total number of items is 31, so according to Hair et al. (2011), there must be a minimum of 10 respondents against each item. Therefore, to generalize the results on the commodity market investors, 370 questionnaires were distributed to investors.

### Data Collection

The questionnaires were distributed among the investors in the commodity market of developing countries. A pilot study is conducted to check the content and face validity of the instrument. The questionnaire is distributed to the 30 respondents, and the Ph.D. faculty/behavioral experts and stock market investors are involved in checking the content and face validity. The experts edited the following items: 2nd item, emotional intelligence; 5th item, decision-making; and 3rd item, optimism bias. After incorporating the changes, the final questionnaire (Table 1) is distributed among 370 respondents, of which, 15 questionnaires were discarded as the respondents did not answer the questionnaires. Eighteen questionnaires were not received from the respondents. The final 337 respondents’ data were entered in the SPSS. The final response rate was 91.08. In addition, due to the time factor, cost factor, and easy accessibility (respondents are not easily available), the convenience base sampling method is used as this method provides the highest response level while saving resources and timely feedback (Etikan et al., 2016).

**TABLE 1:** Breakdown of questionnaire.

Composition of questionnaire		

Particulars	No. of questionnaires	Percentage (%)
Questionnaires distributed	370	
Questionnaire completed	337	91.08
Questionnaire discarded	18	4.86
Questionnaire not received	15	4.05

*Total 370 questionnaires are distributed and 337 respondents’ data are entered in the SPSS 26.*

### Measurement

Questionnaires are distributed to the respondent of the commodity market. Data were collected from the five-point Likert scale as 1 depicts “strongly disagree” and 5 indicates “strongly agree.” Emotional intelligence is measured by the scale of Davies et al. (2010) consisting of 10 items. A sample item is “I know why my emotions change.” Decision-making is the outcome variable, and it is also measured on the Likert scale proposed by Scott and Bruce (1995) consisting of 5 items related to the irrational behavior of the investor. A sample item of decision-making is “When making an investment, I trust my inner feelings and reactions.” Optimism is a first-order mediating variable consisting of 12 items, based on the 5-point Likert scale from strongly disagree to strongly agree. A sample item of optimism bias was “In uncertain times, I usually expect the best,” which was developed by Scheier and Carver (1985). The risk perception is a second-order mediating variable consisting of 4 items. A sample item of risk perception is “I invest 10% of my annual income in moderate growth securities,” which was developed by Weber et al. (2002).

### Reliability Analysis

Cronbach’s alpha is a statistical formula that is used to measure the internal consistency between the items. It is a very useful model fit that measures if the scale is perfectly good or not. According to Tavakol and Dennick (2011), the minimum allowed value under the Cronbach’s alpha is 0.70, and if the value is lower than that, then the reliability of scale (internal consistency) is not a model fit. The results of this study indicated that Cronbach’s alpha values of all the variables are in the satisfactory range (Table 2).

**TABLE 2:** Reliability statistics.

	Cronbach’s alpha	No. of items
Decision-making	0.712	05
Emotional intelligence	0.810	10
Optimism bias	0.830	12
Risk perception	0.753	04

*Cronbach’s alpha >0.70; satisfactory.*

### Demographic Variables

Descriptive statistics (Table 3) showed the respondents’ age, gender, and education characteristics. Results reported that the maximum number of investors involved in the commodity market’s trading activity is 34.42% and the minimum number of investors more than 40 years is 7.71%. In addition, results also indicated that the male respondents are more interested in investing in the commodity market than the female investors, and the percentage of male respondents was 86.64%. The investors’ financial literacy (knowledge about financial products) is very low. As for the concern of ordinary education, only 29.08% of investors are bachelors.

**TABLE 3:** Descriptive statistics.

	Frequency	Percent
**Age**		
25–30	28	8.30
31–35	167	49.55
36–40	116	34.42
More than 40	26	7.71
**Gender**		
Women	45	13.35
Men	292	86.64
**Education**		
Matric	54	16.02
Intermediate	76	22.55
Bachelors	98	29.08
Masters	82	24.33
Post graduate	27	8.01

*Frequency distribution of respondents; age, gender, and education.*

### Statistical Technique

Regression analysis measures the intensity of predictors on the outcome variable. The statistical technique for mediation is performed in PROCESS macros (Hayes and Scharkow, 2013). Model 6 of PROCESS macro is used to analyze the results as proposed by Hayes (2017). AMOS is used for convergent and discriminant validities along with model fit analysis. It is a calculating technique that is used to analyze the conditional effect under the SPSS statistical technique.

In Table 4, validity is performed and checked through the composite reliability (CR), average variance extracted (AVE), and maximum shared variance (MSV). The reliability values for all variables fell within the acceptable range of 0.7–0.9 (Hair et al., 2011). In addition, for convergent validity, the AVE score is greater than 0.5, which is in the acceptable range (Fornell and Larcker, 1981), and the MSV value is also less than the AVE value. The square root of AVE also determines that the validity must be greater than its paired correlation.

**TABLE 4:** Validity.

	CR	AVE	MSV	EI	OB	RP	DM
EI	0.912	0.776	0.604	**0.881**			
OB	0.897	0.637	0.224	0.07	**0.798**		
RP	0.87	0.532	0.438	0.064	0.474	**0.729**	
DM	0.925	0.756	0.604	0.777	0.121	0.048	**0.87**

n = 337. Bolded values on the diagonals are the square root values of AVE. AVE, average value extracted; MSV, maximum shared variance; CR, composite reliability; EI, emotional intelligence; OB, optimism bias; RP, risk perception; DM, decision-making.

Table 5 depicts the model fit analysis, namely, absolute fit, incremental fit, and parsimonious fit (Hair et al., 2011). According to Hu and Bentler (1999), the RMSEA value must be near 0.05 and not more than 0.08. Researchers have proposed three categories of fit indexes: absolute, incremental, and parsimonious fit (Hu and Bentler, 1999; Hair et al., 2011). It has also been proposed that at least one fit index from each category must be included to confirm a model’s fitness. Table 5 presents the threshold level and model fit indexes, with the data showing that the structural model fits the data as CMIN/DF = 2.132, GFI = 0.908, CFI = 0.92, 0.943, NFI = 0.901, PCFI = 0.806, and PNFI = 0.764 so as to meet the criteria of the threshold level.

**TABLE 5:** Model fit.

Category	Indexes	Value of index	Threshold level
Absolute fit	RMSEA	0.062	<0.06
	CMIN/DF	2.132	<3.0
	GFI	0.908	>0.90
Incremental fit	CFI	0.921	>0.90
	TLI	0.943	>0.90
	NFI	0.901	>0.90
Parsimonious fit	PCFI	0.806	>0.50
	PNFI	0.764	>0.50

Table 6 depicts predictors’ direct and indirect effects on the outcome variable. The results showed that emotional intelligence has a significantly positive (*p* < 0.05) effect on decision-making and that the influence factor of emotional intelligence on decision-making is 25.3%. Moreover, β = 0.273 indicated that the positive change occurs in the outcome variable (decision making) by a change in emotional intelligence. The second hypothesis investigated the effect of risk perception on decision-making. The results (*p* < 0.05, β = 0.191) indicated that risk perception has a positive significant effect on decision-making. The effect of optimism bias on decision-making (third hypothesis) was also investigated. The results showed a significant positive effect of optimism bias on decision-making. Moreover, *R*^2^ = 0.048 indicates the effect of optimism bias on decision-making as 4.8%. The fourth hypothesis investigated the effect of emotional intelligence on optimism bias. The results showed a significant effect of emotional intelligence on optimism bias. The fifth and sixth hypotheses (emotional intelligence and optimism bias effect to risk perception) were also investigated by the regression effect; both predictors had a significant (*p* < 0.05) effect on risk perception. The unstandardized value showed the positive effect on risk perception, i.e., β = 0.221 and β = 0.122. Model 6 of PROCESS macro was applied to examine the effect of emotional intelligence on decision-making *via* the intervening effect of optimism bias and risk perception. The results (Table 7) showed a significant effect of the intervening variables between emotional intelligence and decision-making. Moreover, it was also observed that there is a partial mediation between emotional intelligence and decision-making *via* the mediating effect of optimism bias and risk perception, respectively.

**TABLE 6:** Model summary.

Model	*R*	*R* ^2^	Sig	Unstandardized coefficients
Emo → DM	0.503	0.253	0.000	0.273
RP → DM	0.428	0.183	0.019	0.191
OPT → DM	0.219	0.048	0.000	0.295
Emo → OPT	0.436	0.190	0.000	0.140
Emo → RP	0.563	0.316	0.000	0.221
OPT → RP	0.372	0.138	0.000	0.122
Emo + OPT → RP → DM	0.4475	0.200	0.000	0.235

*Regression analysis; significant, if p < 5%; unstandardized coefficient* = β.

**TABLE 7:** Model summary.

Hypotheses	Relationship/effect	Accepted/rejected
H1	Emo → DM	Accepted
H2	RP → DM	Accepted
H3	OPT → DM	Accepted
H4	Emo → OPT	Accepted
H5	Emo → RP	Accepted
H6	OPT → RP	Accepted
H7	Emo → OPT → RP → DM	Accepted

*Emo, emotional intelligence; DM, decision-making; RP, risk perception; OPT, optimism bias.*

## Discussion

Behavioral finance researchers state that psychological feelings influence an individual’s decisions and they are irrational (Nguyen and Rozsa, 2019). Behavioral finance’s psychology and theories identified deviations from standard finance. The laypeople have no information about irrational behavior (Mellios et al., 2016). This study analyzes the serial mediation effect, namely, optimism bias and risk perception between emotional intelligence and investment decisions in the commodity market.

Hypothesis 1 stated the effect of emotional intelligence on investment decisions. The results revealed that emotional intelligence significantly affects investment decisions and aligns with the previous literature as Mayfield et al. (2008) also examined that emotional intelligence has a strong relationship with virtue and investment performance. Thus, the prospect theory is applied to this hypothesis as cognitive psychology influences decision-making rather than market information. Hypothesis 2 examined the effect of risk perception on decision-making. Nguyen and Rozsa (2019) observed that risk perceptions mediate the relationship between cognitive biases and investment decisions. Hypothesis 3 stated that emotional intelligence significantly affect risk perception. The results of this hypothesis are also aligned with the prospect theory because of the effect of uncertainty (risk) during decision-making. The results (β = 0.221, *p* < 5%) revealed a significant effect of emotional intelligence on risk perception. Hypothesis 4 stated that emotional intelligence significantly affects risk perception. Campos and Keltner (2014) also examined that emotional intelligence strongly relates to virtue and investment performance under risky investment. Hypotheses 5 also supported the effect of emotional intelligence on risk perception. Wang et al. (2011) also reported that emotional intelligence significantly affects risk perception. Hypothesis 6 stated that optimism bias significantly affects risk perception. The results (β = 0.122) also reported that optimism bias positively affects risk perception. This hypothesis also contradicts traditional finance due to the effect of optimism bias (judgment error) in the decision-making process. The behavioral finance reported that cognitive biases are involved in the decision-making process *via* the mediating role of risk perception. Ingram et al. (2019) also examined the mediating effect of risk perception between cognitive biases and decision-making on venture capital.

This study will help the investors understand an investor’s behavioral effect. Moreover, this study will support commodity market investors and potential investors by highlighting their behavioral biases and the importance of risk perception. Several kinds of research have examined behavioral biases and their effects on decision-making. However, relatively little has been written about the relationship between investment decisions and behavioral bias in the commodity market. Vasileiou (2021) identified the gap that, although rational decision affects investment decisions, fewer investigations have been made on behavioral finance. Given the scarcity of research on the intervening mechanisms (optimism bias and risk perception) of the relationship between emotional intelligence and decision-making, the present study is timely and relevant.

## Conclusion

Economic, political, and behavioral changes influence an investor’s buying and selling patterns. However, share market prices are affected by global economic fluctuations. Investors’ decisions are influenced by various factors, including changes in share prices caused by different circumstances. Most investors are irrational in business activities (Pfnür and Wagner, 2020). They invest in stocks only based on their previous experience. They do not understand why share prices are reflected or vary due to the lack of business knowledge. A financial analyst who is both a chartered accountant and a CFA analyst is hired by many investors. Business students have a rudimentary understanding of how practical decisions are made and how the market fluctuates.

### Practical Implications

Timely decisions are always a key element for investors. Various factors affect the investment decisions through the market information and judgment errors called cognitive biases. Meanwhile, the literature also addressed that managers’ decisions are based on personality types and emotional intelligence. This study is very timely and relevant as the investors must consider that, rather than the market, their emotional intelligence also influences decision-making. The present study results will improve the investors’ decisions by combining emotional intelligence and market information. This study will also be very helpful for the educational institutions as they can revise the course curriculum by keeping in mind the traditional and classical financial decisions. In addition, most investors rely on brokerage houses for decision-making. Investors must show their keen interest in the decision-making by considering their emotional intelligence, optimism bias, and risk perception. This study is also crucial regarding the institutional/financial houses as they are internationally involved in the commodity market.

### Theoretical Implications

This study has numerous theoretical contributions in the literature on the irrational decision-making of investors. First, complicated decisions are based on investors’ intuition, perceptions, emotions, and thinking (Kahneman and Riepe, 1998). Still, these decisions are often irrational as cognitive biases are involved and complete information is ignored. So, this study focuses on investigating the emotional intelligence of an investor and its effect on decision-making. Second, the study highlights the impact of optimism bias on the investor’s decisions. As Costa et al. (2017) examined, investors’ decisions are based on irrational behavior rather than market information. Investors follow the optimism approach when they are positive about their confidence. Based on positive thoughts as well as reality, optimistic investors make decisions. Some investors allocate the resources in a short-term period and others go for the long-term investment (Englmaier, 2010). These investment opportunities also vary with the change in economic situations. Investors get optimal satisfaction in both types of investment by utilizing the best investment decision (Sattar et al., 2020). Third, the prospect theory proposes that investors’ decisions are based on the uncertainty factor called risk. So, this study contributes to the body of knowledge by studying risk perception as a mediating variable between optimism bias and decision-making. Still, limited studies address investors’ behavioral aspects in the commodity market; investors are unaware that they rely too much on the managers and depend upon them. If investors have high emotional intelligence, they can better perceive the risk and make better decisions. Unfortunately, in the commodity market, these factors are ignored, so this study contributes to the literature by studying the emotional intelligence, optimism bias, and risk perception of an investor.

### Limitations and Future Recommendations

This research study considers the commodity market investors with the behavioral aspect. Time-lag study must be conducted to analyze investor behavior. Moreover, future studies can be performed on the stock exchange and real estate sector. This study only considers the quantitative approach, uses a close-ended questionnaire, and ignores the qualitative approach through interviews (open-ended questions). The respondent’s feedback can be utilized to make better decisions through qualitative research. Data were gathered from investors and only focused on the commodity market and may not be considered by the other segments of the population. Future studies on the comparative study of commodities and stock exchange investors can be done. In addition, future research can also explore the real-estate segment with the investor’s behavior. Financial literacy plays a vital role in decision-making, so future studies should be made on the moderating effect of financial literacy between cognitive biases and decision-making. Moreover, investors’ decisions can be changed under the short- and long-term investment intentions. So, future studies can be done under short- and long-run periods.

## Data Availability Statement

The original contributions presented in the study are included in the article/supplementary material, further inquiries can be directed to the corresponding author.

## Ethics Statement

The studies involving human participants were reviewed and approved by the Management Sciences Department of Riphah University. The patients/participants provided their written informed consent to participate in this study.

## Author Contributions

CC identified the problem statement. MI filled the research gap and developed hypotheses. FA wrote the introduction section. AS wrote the methodology. KW wrote the result discussion and conclusion. All authors listed have made a substantial, direct, and intellectual contribution to the work, and approved it for publication.

## Conflict of Interest

The authors declare that the research was conducted in the absence of any commercial or financial relationships that could be construed as a potential conflict of interest.

## Publisher’s Note

All claims expressed in this article are solely those of the authors and do not necessarily represent those of their affiliated organizations, or those of the publisher, the editors and the reviewers. Any product that may be evaluated in this article, or claim that may be made by its manufacturer, is not guaranteed or endorsed by the publisher.
